# Radiologic follow-up in Fontan-associated liver disease in Europe: European Society of Paediatric Radiology survey demonstrates the need for a consensus protocol

**DOI:** 10.1007/s00247-021-05172-y

**Published:** 2021-10-16

**Authors:** Giulia Perucca, Charlotte de Lange, Stéphanie Franchi-Abella, Marcello Napolitano, Michael Riccabona, Damjana Ključevšek, Seema Toso, Jochen Herrmann, Samuel Stafrace, Kassa Darge, Maria Beatrice Damasio, Costanza Bruno, Magdalena Maria Woźniak, Luisa Lobo, Donald Ibe, Anne M. Smets, Philippe Petit, Lil-Sofie Ording Müller

**Affiliations:** 1 Department of Pediatric Radiology, Regina Margherita Children’s Hospital, Turin, Italy; 2 Department of Radiology and Clinical Physiology, Queen Silvia Children’s Hospital, Sahlgrenska University Hospital, Göteborg, Sweden; 3 Pediatric Radiology Department, Hôpital Bicêtre, Hôpitaux Universitaire Paris-Sud, Assistance Publique Hôpitaux de Paris, Le Kremlin-Bicêtre, France; 4 Department of Paediatric Radiology and Neuroradiology, V. Buzzi Children’s Hospital, Milan, Italy; 5 Department of Radiology, Division of Pediatric Radiology, Medical University Graz and University Hospital LKH, Graz, Austria; 6 Department of Radiology, University Children’s Hospital Ljubljana, Ljubljana, Slovenia; 7 Department of Pediatric Radiology, University Hospital of Geneva, Geneva, Switzerland; 8 Department of Pediatric Radiology, University Hospital Hamburg Eppendorf, Hamburg, Germany; 9 Department of Diagnostic Imaging, Sidra Medicine, Doha, Qatar; 10 Weill Cornell Medicine, Doha, Qatar; 11 Department of Radiology, The Children’s Hospital of Philadelphia, University of Pennsylvania, Philadelphia, PA USA; 12 Radiology Department, IRCCS Istituto Giannina Gaslini, Genoa, Italy; 13 Department of Radiology, Azienda Ospedaliera Universitaria Integrata Verona (AOUI), Verona, Italy; 14 Department of Pediatric Radiology, Medical University of Lublin, Lublin, Poland; 15 Serviço de Imagiologia Geral, Hospital de Santa Maria–Centro Hospitalar Universitário Lisboa, Norte (CHULN), Lisbon, Portugal; 16 Department of Radiology, Silhouette Diagnostic Consultants, Abuja, Nigeria; 17 Department of Radiology and Nuclear Medicine, Amsterdam UMC, University of Amsterdam, Amsterdam, the Netherlands; 18 Aix Marseille Université, AP-HM, Equipe d’Accueil 3279 - IFR 125, Hôpital Timone Enfants, Service d’Imagerie Pédiatrique et Prénatale, Marseille, France; 19 Unit for Paediatric Radiology, Department of Radiology, Oslo University Hospital, Rikshospitalet, PB 4950 Nydalen, 0424 Oslo, Norway

**Keywords:** Adolescents, Children, Cirrhosis, Fontan procedure, Hepatocellular carcinoma, Liver, Liver fibrosis, Magnetic resonance imaging, Ultrasound

## Abstract

Fontan surgery is a life-saving procedure for newborns with complex cardiac malformations, but it originates complications in different organs. The liver is also affected, with development of fibrosis and sometimes cirrhosis and hepatocellular carcinoma. There is no general agreement on how to follow-up these children for the development of liver disease. To understand the current practice on liver follow-up, we invited members of the European Society of Paediatric Radiology (ESPR) to fill out an online questionnaire. The survey comprised seven questions about when and how liver follow-up is performed on Fontan patients. While we found some agreement on the use of US as screening tool, and of MRI for nodule characterization, the discrepancies on timing and the lack of a shared protocol make it currently impossible to compare data among centers.

## Introduction

The Fontan procedure is a lifesaving palliative multi-step surgery performed in children with complex cardiac malformations who have a single functional ventricle [1]. Complications are, unfortunately, multiple because of the modified circulation as well as the damage occurring in the prenatal and preprocedural period. These complications appear in most pediatric Fontan patients [2]. Liver complications have recently gained more attention and include fibrosis, often leading to cirrhosis. Hepatocellular carcinoma can also develop, even at an early age [3]. Because it has a peculiar pathophysiology and is not entirely understood, Fontan-associated liver disease is considered a distinct entity from other causes of liver fibrosis and cirrhosis.

To detect significant liver changes and malignancy at an early stage, Fontan patients need careful follow-up based on imaging. However, there is no established surveillance protocol for liver imaging concerning methods or time intervals, which makes it impossible to compare data among centers.

Some imaging algorithms for Fontan-associated liver disease follow-up have recently been proposed in North America [4, 5]. Our experience suggests that the current practice in Europe might differ. To understand how Fontan-associated liver disease is monitored in Fontan patients in Europe, and with the long-term aim of making our practice more homogeneous and comparable, we set up a survey among members of the European Society of Paediatric Radiology (ESPR).

## Survey

We sent an online survey to all 489 ESPR members using Google Forms via repeated newsletters from January 2020 to May 2020 because of an initial low response rate. The survey comprised seven questions: the first documented the name of the respondent’s hospital, to avoid duplicates; the others assessed whether the Fontan procedure was performed in the respondent’s institution, whether liver follow-up was performed and how, whether anything was added to the basic protocol in cases of new liver nodules measuring more than 10 mm.

The questions in detail were as follows:

1. What is the name of your hospital? (Free text)
2. Do you perform liver imaging on patients with Fontan circulation in your hospital? (Yes/No)
3. Is the surgical Fontan procedure performed in your hospital? (Yes/No)
4. If you perform liver imaging of patients with Fontan circulation, what do you routinely do? (Several answers are possible: Abdominal US with elastography, Abdominal US without elastography, CEUS [contrast-enhanced ultrasound], Liver MR with hepatospecific contrast agent, Liver MR without hepatospecific contrast agent, Liver MR without injection, Liver MR elastography)
5. At what age (in years) is liver follow-up started? (Free text)
6. How often is liver follow-up performed (in months)? (Free text)
7. What else do you add to the normal follow-up in case of new finding of liver nodules >10 mm in diameter? (Nothing, the patient undergoes the usual follow-up / Abdominal US with elastography / Abdominal US without elastography / Liver MR with hepatospecific contrast agent / Liver MR without hepatospecific contrast agent / Liver MR without injection / Liver MR elastography)

## Responses

We received responses from 24 European hospitals (4.9% of members), and 1 United States and 1 Canadian hospital. Because of the aim of the survey, only responses from the European centers were included in the analysis.

### Centers performing Fontan-associated liver disease follow-up

Fontan procedure was performed in 14 of the responding institutions (58.3%). Of those, 9/14 (64.3%) performed liver imaging as part of the follow-up for Fontan patients. In the other 10 hospitals where Fontan surgery was not performed, liver imaging was done anyway for Fontan patients in 6 centers (60%), so that liver imaging was overall performed in 15/24 centers (62.5%).

### Routine Fontan-associated liver disease follow-up

**Fig. 1:**
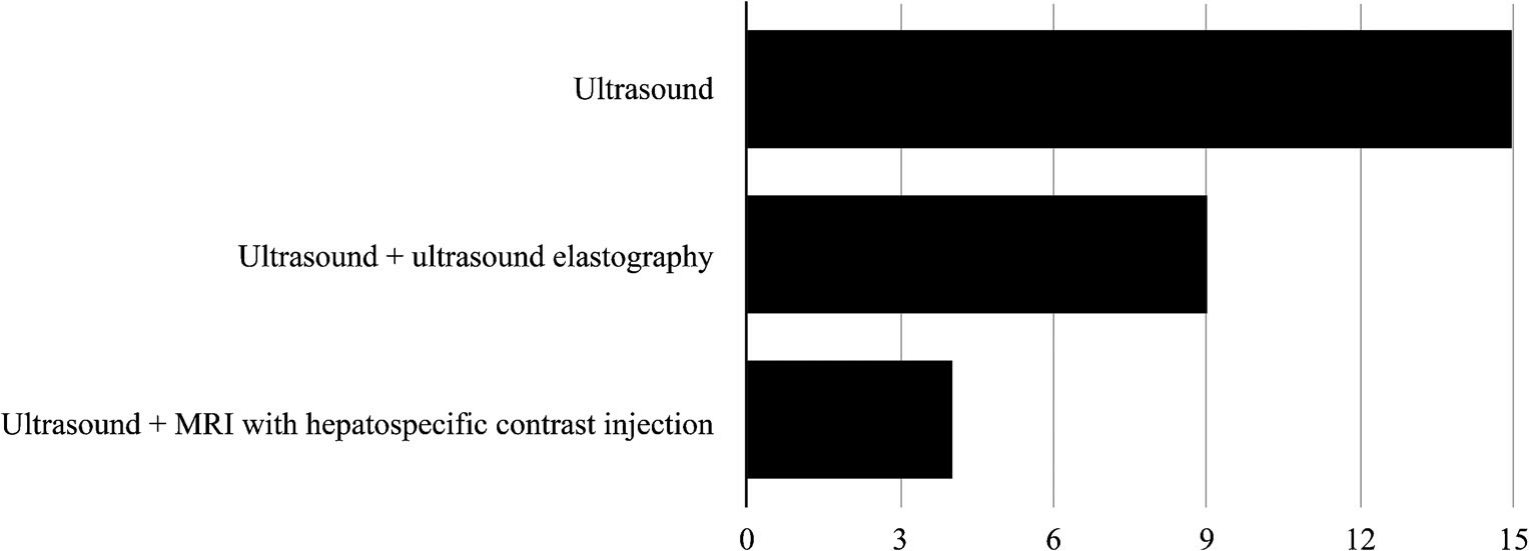
Graph shows imaging techniques routinely performed at the 15/24 centers that reported doing liver follow-up for Fontan-associated liver disease

Abdominal US was reported as always being part of the basic follow-up (15/15, 100%), with the addition of elastography at 9/15 (60%) institutions. At 4/15 (27%) institutions, MRI was also performed routinely, with hepatobiliary contrast agent used in all (4/4, 100%) of them (Fig. 1).

### Patient age for beginning Fontan-associated liver disease follow-up

**Fig. 2:**
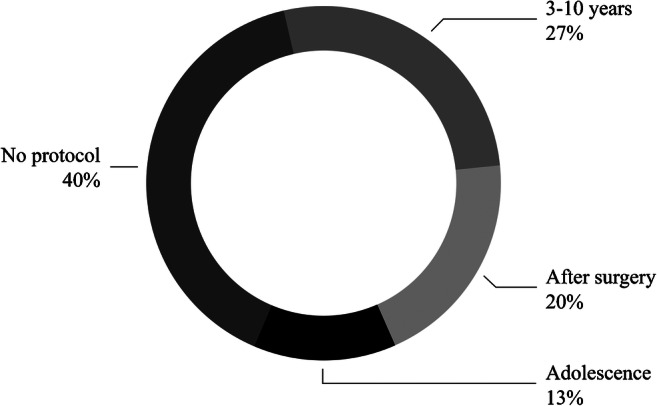
Graph shows patient age at the start of follow-up for Fontan-associated liver disease at different centers. Forty percent of the respondents reported that they did not have a protocol at their institution

Patient ages at the initiation of follow-up were extremely heterogeneous. Three of 15 (20%) institutions began just after Fontan completion. At 4/15 (27%) institutions, imaging started during childhood (age range 3–10 years at different centers), whereas at 2/15 (13%) institutions it began when children were in their teens. Respondents from the remaining 6/15 (40%) institutions that performed follow-up reported that their institutions did not have a protocol (Fig. 2).

### Frequency of Fontan-associated liver disease follow-up

**Fig. 3:**
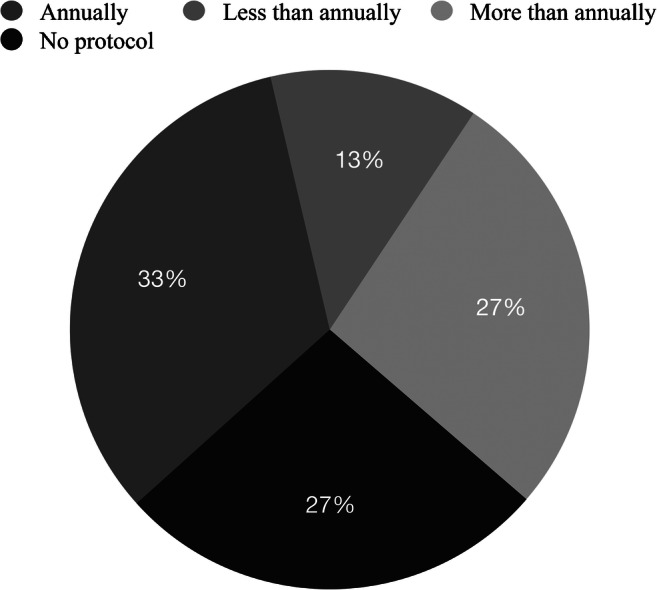
Pie chart shows frequency of follow-up for Fontan-associated liver disease at different centers. Among respondents whose institutions performed follow-up imaging, 4/15 (27%) reported not having a protocol at their institution

Regarding how frequently liver imaging is performed in the 15 centers that reported follow-up for Fontan-associated liver disease: 5 (33%) centers followed up annually, 4 (27%) centers more frequently (every 3–6 months or every 8 months), 2 (13%) centers less frequently (every 2 years or 3–5 years). Four of the 15 (27%) did not know the protocol or had no protocol (Fig. 3).

### Additional imaging for nodules >10 mm

**Fig. 4:**
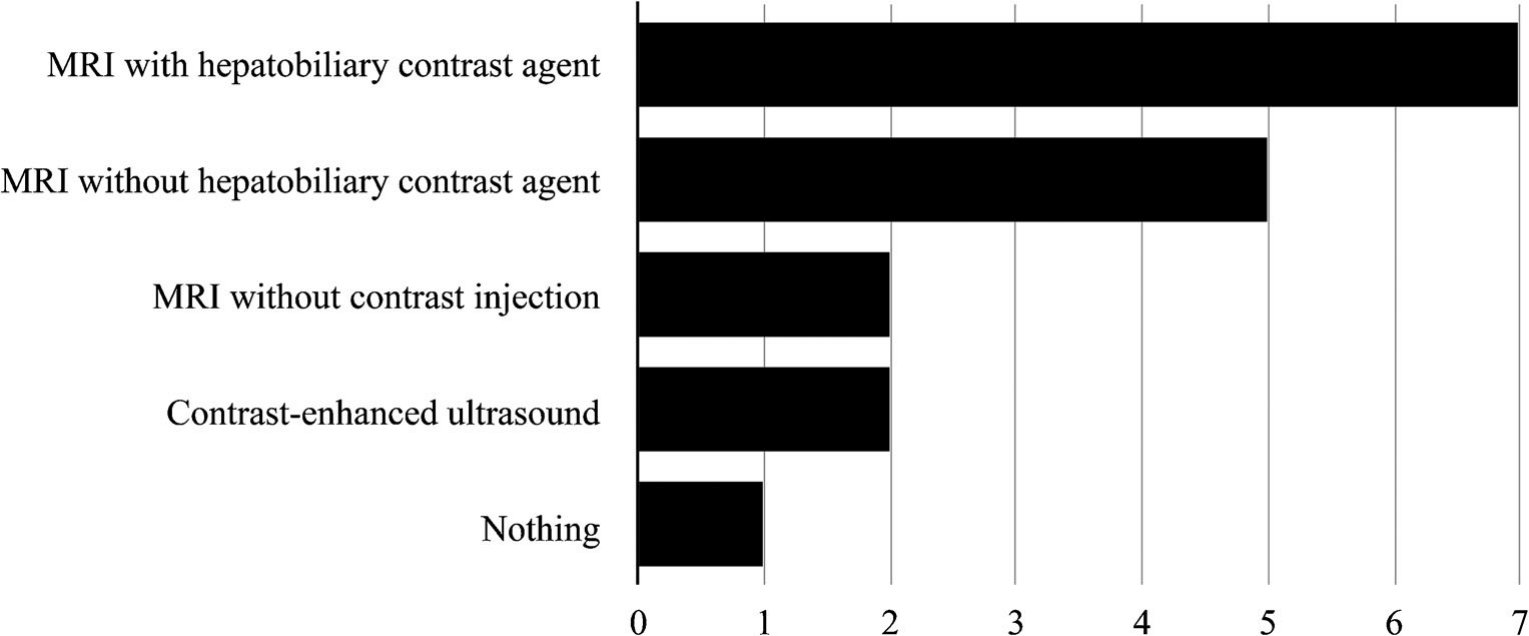
Graph shows additional imaging performed at different centers in cases of a new nodule >10 mm found at routine US

In cases of a new finding of a liver nodule >10 mm, we asked respondents what was added to the normal protocol. At one institution, nothing was added to the basic protocol; however, that institution already included US, US elastography and liver MR with hepatobiliary contrast agent as routine. MRI was performed at all other institutions if a nodule of >10 mm was found at basic follow-up, with hepatobiliary contrast agent at 7/15 (47%) institutions, without hepatobiliary contrast agent at 5/15 (33%) institutions and without gadolinium contrast injection at 2/15 (13%) institutions. In addition to MRI, two institutions also performed contrast-enhanced ultrasound (CEUS) in such cases (Fig. 4).

## Discussion

This study demonstrates that among European pediatric radiology departments practice varies about when to start follow-up for Fontan-associated liver disease and how frequently to perform it. This is not surprising because very little guidance is available in the literature.

Interestingly, there was 100% agreement on the imaging modality used for part or all of the basic follow-up. In fact, every center performed US as the basic screening tool. Also, there was complete agreement on performing MRI in cases of a suspicious nodule being found on US. These agreements could be derived from the radiologists’ experience in liver pathology of various etiologies, in which the advantages of US as a screening tool and MRI for liver nodule characterization have been demonstrated. However, different centers tend to use different contrast agents. This might be explained by different policies and local availability.

This study has some limitations. The survey was addressed to ESPR members only and received a small number of responses in comparison to other ESPR surveys [6, 7]. This probably reflects, at least in part, the small number of pediatric radiologists involved in liver imaging for Fontan-associated liver disease follow-up. Also, this could correspond to a lack of knowledge, even in tertiary and quaternary centers, on the possibility for Fontan patients to develop malignancy. Thus, these children might not be referred to radiology for routine investigation.

## Conclusion

This survey highlighted the need for a consensus protocol for liver imaging follow-up in Fontan patients. The relative agreement on usefulness of US as a screening tool in liver fibrosis and cirrhosis and of MRI in liver nodule characterization might be the basis for the development of a shared protocol. The Abdominal Task Force of the ESPR plans to take this work forward to develop a consensus-based follow-up algorithm.
